# The impact of adding an extra dimension to a preference-based measure

**DOI:** 10.1016/j.socscimed.2011.05.026

**Published:** 2011-07

**Authors:** John Brazier, Donna Rowen, Aki Tsuchiya, Yaling Yang, Tracy A. Young

**Affiliations:** a University of Sheffield, UK; b Brunel University, UK

**Keywords:** Preference-based measures of health, Cross programme comparability, Condition-specific measures, UK

## Abstract

The ability to compare incremental changes in Quality Adjusted Life Years (QALYs) generated by different condition-specific preference-based measures (CSPBMs), or indeed between generic measures, is often criticised even where the valuation methods and source of values are the same. A key concern is the impact of excluding key dimensions from a descriptive system. This study examines the impact of adding a generic pain/discomfort dimension to a CSPBM, the AQL-5D (an asthma-specific CSPBM), by valuing samples of states from the AQL-5D with and without the new dimension using an interviewer administered time trade-off with a sample of the UK general public. 180 respondents provided 720 valuations for states with and without pain/discomfort. As expected the additional pain/discomfort dimension was found to have a significant and relatively large coefficient. More importantly for comparing changes in QALYs across populations the addition of pain/discomfort significantly impacts on the coefficients of the other dimensions and the degree of impact differs by dimension and severity level. The net effect on the utility value depends on the severity of their state: the addition of pain/discomfort at level 1 (no pain/discomfort) or 2 (moderate pain/discomfort) significantly increased the mean health state values in an asthma patient population; whereas level 3 pain/discomfort (extreme) reduced values. Comparability between measures requires that the impact of different dimensions on preferences is additive, whether or not they are included in the classification system. Our results cast doubt on this assumption, implying that the chosen measure must contain all important and relevant dimensions in its classification system.

## Highlights

► As expected the additional pain or discomfort dimension was associated with significant coefficients for levels 2 and 3 in the model of the health state valuation data. ► The additional dimension had a significant impact on the coefficients of other dimensions, but this differed by dimension and level. ► The net impact on the health state value depended on the level of the pain or discomfort dimension, with level 1 or 2 being associated with higher values and level 3 with lower values. ► This has important implications for the strategy of adding dimensions to existing instruments and for the achievement cross instrument comparability.

## Introduction

Recent years has seen the rise of generic preference-based measures in populating cost per QALY analyses, with the EQ-5D gaining a special status as the preferred measure for economic evaluations submitted to the National Institute for Health and Clinical Excellence of England and Wales (NICE, 2008). It has been claimed that ‘generic’ preference-based measures are applicable to all interventions and patient groups. This claim has support in many conditions where it has been shown to be reliable, valid and responsive (Brazier, Ratcliffe, Tsuchiya, & Solomon, 2007). However, one or more of the generic preference-based measures have been shown to perform poorly in some conditions, such as visual impairment in macular degeneration (Espallargues et al, 2005), hearing loss (Barton, Bankart, & Davis, 2004), leg ulcers (Walters, Morrell, & Dixon, 1999), and urinary incontinence (Haywood, Garratt, Lall, Smith, & Lamb, 2008). For this and other reasons many clinicians and researchers use condition-specific measures that are not preference-based.

There has been increasing interest in the development of condition-specific preference-based measures (CSPBM). This has been achieved either by the development of entirely new measures (e.g. Revicki, Leidy, Brennan-Diemer, Sorenson, & Togias, 1998; Revicki, Leidy, Brennan-Diemer, Thomson, & Togias, 1998; Torrance et al., 2004), or by developing health state classifications amenable to valuation from existing condition-specific measures (Brazier et al., 2008; Kok, McDonnell, & et al, 2002; Ratcliffe, Brazier, Tsuchiya, & et al, 2009; Stevens, Brazier, McKenna, Doward, & Cork, 2005; Young, Yang, Brazier, Tsuchiya, & Coyne, 2009). However, there remain some fundamental concerns as to whether they can be used to make comparisons between interventions for different conditions (Brazier & Tsuchiya, 2010; Dowie, 2002; Gold, Siegel, Russell, & Weinstein, 1996). Even using generic systems does not ensure comparability, since significant differences have been shown between the different generic preference-based measures (Moock & Kohlmann, 2008). One way to achieve cross programme comparability is to use the same generic preference-based measure. Using one instrument in all studies ensures that different patient groups are being judged in terms of the same dimensions of health, using the same valuation methods and utility values obtained from the same sample. For this reason, NICE has expressed a preference for the EQ-5D in its reference case methods (NICE, 2008), though other agencies interested in seeing cost utility analysis of health care interventions have been less prescriptive.

An alternative view is that comparability can be achieved by the use of a common numeraire like money or a year in full health. Provided the values are obtained using the same tightly specified valuation ‘protocol’ in terms of the valuation technique (and variant), procedures, common anchors (full health and death), visual aids and the same type of respondents (such as a representative sample of the general population), then a common measuring stick is being used and so comparisons can be made between quality adjustment weights estimated using different descriptive systems. This means that there is no need to have a common descriptive system.

However, there are a number of obstacles to achieving comparability from using different descriptive systems including the need to handle side-effects and co-morbidities (Brazier & Tsuchiya, 2010). The failure to pick-up important side-effects of treatment is the rationale in clinical research for using a generic measure alongside a condition-specific measure in a trial. The problem for economic evaluation is that it needs a single measure of effectiveness. Even assuming there are no side-effects, the achievement of comparability between specific preference-based instruments requires an additional assumption, namely that the impact of different dimensions on preferences is additive, whether or not they are included in the descriptive system. The impact of breathlessness on health state values, for example, must be the same whether or not the patient has co-morbidities that impact on dimensions not covered by the descriptive system, such as pain in joints.

The impact of dimensions external to the descriptive system may be the product of a form of focussing effect (or focussing illusion). We focus on those things that are placed in front of us. Respondents, therefore, will tend to focus on the problems described in the health state they are valuing. This results in respondents exaggerating the importance of the problems in the health state they are being asked to value to the neglect of any domains not covered by the health state classification system. They may have a view about the level of other dimensions, but this may carry less weight. Or alternatively, different people may bring different assumptions about the level of the unmentioned dimensions. Either way, the addition of dimensions may have implications for the entire structure of the utility function for health.

This issue has implications for the development of add-on dimensions to extend the coverage of generic measures like the EQ-5D. Studies have examined the impact of adding-on dimensions for cognition (Krabbe, Marlies, Stouthard, & et al, 1999) and sleep (Yang et al, 2008), which in the case of the former was found to be significant in a student population and in the latter not significant in a sample of the general population using time trade-off (TTO). The same approach can be applied to improving CSPBM that are narrower in their focus by adding-on more generic dimensions. This paper examines the impact of adding-on a pain/discomfort dimension to a preference-based asthma-specific measure. Pain/discomfort has been shown to have a large impact on health state values across the generics, so it provides a good opportunity to test the concerns with CSPBM raised above as well as the more general problems associated with the add-on approach.

The main aim of this study is to examine the impact of adding a generic pain/discomfort dimension to a CSPBM for asthma and specifically to test whether the impact on health state values is additive. This is achieved by asking a general population sample to value a selection of health states defined by AQL-5D (an asthma-specific health state classification) or AQL-6D (AQL-5D plus pain/discomfort dimension) using TTO. Coefficients for the 5 and 6 dimensional classifications are estimated and the coefficients of the common dimensions are compared. The impact on health state values of using AQL-5D or AQL-6D is examined using data from a randomised controlled trial.

## Methods

### Measures of health-related quality of life: AQL-5D and AQL-6D

The study uses the AQL-5D, which is a 5-dimension 5-level preference-based measure for asthma (Yang et al, 2011; Young et al, 2011). The health state classification system was derived from the Asthma Quality of Life Questionnaire (AQLQ) (Juniper, Guyatt, Ferrie, & Griffith, 1993, 1999) using Rasch and conventional psychometric analysis (Young et al, 2011). The 5 dimensions are: concern about asthma, shortness of breath, weather and pollution stimuli, sleep impact and activity limitations. In the classification system each dimension has 5 levels of severity with level 1 denoting no problems and level 5 indicating extreme problems. All patient data with complete AQLQ information can be mapped onto the AQL-5D. The original valuation study selected 99 health states for valuation using a balanced design. States were then valued using the Measurement and Valuation of Health (MVH) study version of TTO, which includes a visual prop (Dolan, 1997; Gudex, 1994). The preference weights for all states defined by the classification model were estimated using a consistent main effects model estimated on mean health state values (Yang et al, 2011).

In the study reported in this paper, a reduced AQL-5D health state classification system is valued where each dimension has 3 levels of severity: level 1 denoting no problems, level 2 denoting some problems and level 3 denoting extreme problems (see Table 1). These relate to levels 1, 3 and 5 in the original classification system. The reduced classification was chosen primarily to limit the size of the valuation survey required to address the study aim. The selection of the 3 levels also makes sense because in the original valuation study level 2 was insignificant for all dimensions in the regression models estimating the preference weights for the classification system. Level 4 was significant for all dimensions but there were inconsistencies between levels 4 and 5 for 2 dimensions (shortness of breath and activity limitations) and for all dimensions the difference in coefficients between levels 4 and 5 was small.

The AQL-6D is a classification system consisting of the 5 dimensions of the reduced AQL-5D plus the pain/discomfort dimension from the EQ-5D added at the end, which also has 3 levels (Brooks, 1996 (see Table 1). This extra dimension was chosen to ensure little overlap and correlation with the existing dimensions whilst ensuring the additional dimension was able to capture potential co-morbidities and/or side-effects. One advantage of using a dimension from an existing measure is the availability of patient data including both measures (viz. AQLQ and EQ-5D), which enables us to test the impact of adding an additional dimension on health state values using a patient data set from a clinical trial.

### Valuation survey

This study needs to be able to capture the impact on health state values of adding an additional dimension to the AQL-5D classification system, whilst removing the possibility that the observed impact is due to some other factor. Comparing health state values for AQL-6D with the AQL-5D values from the original study (Yang et al, 2011) is unsuitable as any observed differences could be caused by multiple factors, including different samples, different interviewers, the reduction in the number of levels and different techniques used to sample states for the valuation study. Therefore in order to minimise variation for any other reason both the AQL-5D and the AQL-6D were valued by two samples in the same valuation survey.

#### Selection of states

Health states for each measure were selected using an orthogonal array in SPSS version 15. Sixteen health states were selected for AQL-5D, one of which was a repeated state (11111). Eighteen health states were selected for AQL-6D with no repeats. The worst state for each measure (33333 and 333333) was added, taking the number of unique health states to 16 for AQL-5D and 19 for AQL-6D. These included 4 health states that were ‘matched’ across the two descriptive systems in terms of the level of the non-pain dimensions: states 11111 and 1111111; 12132 and 121323; 23131 and 231311; 33333 and 333333. The digits indicate the level in each dimension, so the AQL-5D state 12132 is at level 1 in concern, level 2 in shortness of breath, level 1 in weather and pollution, level 3 in sleep and level 2 in activities. There is no mention of pain/discomfort. The paired AQL-6D state is the same across these asthma-specific dimensions, but there is an explicit reference to pain at the end that in this case is at level 3.

Health states were divided into 3 ‘card blocs’ of 8 states for each measure making 6 blocks or combinations of states in all. The worst state appeared in all card blocks and the remaining matched health states appeared in 2 card blocs to improve power. Other states repeated across more than one bloc for AQL-5D and AQL-6D were chosen to reflect a range of severity (using summed levels and dimensions) and levels for each dimension. Combinations of states within card blocs were chosen to reflect a range of severity (using summed levels and dimensions) and to ensure each card bloc included each level of each dimension. During the interviews, the health state cards were shuffled at the start of the rank and TTO tasks.

Interviewers were instructed to ensure each card bloc was valued equal times per geographical location and to work through the blocs in order with each interviewer starting from a different card bloc. Interviewers were asked to work through the card blocs in order 1 to 6: 1–3 were 5D and 4–6 were 6D. Different interviewers were asked to start with a different bloc first and all blocs were used 3–4 times per geographical area.

#### Respondents

Members of the general population valued 8 health states from either AQL-5D or AQL-6D using time trade-off (TTO). The sampling for all households to be contacted in the study was undertaken using the AFD Names and Numbers version 3.1.25 database for South Yorkshire (AFD Software Limited, Ramsey, UK). This sample was balanced to the UK population according to geodemographic profiles.

#### Interview

All households in the sample were mailed the same information sheet and cover letter, each informing respondents that the interview was concerned with understanding ‘what people think about the way asthma impacts on people’s lives’. Respondents were interviewed in their own home by trained interviewers who have previous experience working on valuation studies including the HUI2 (McCabe, Stevens, Roberts, & Brazier, 2005) and OAB-5D (Yang, Brazier, Tsuchiya, & Coyne, 2009). The interview began with respondents reading and self-completing both the EQ-5D and the AQL-5D, to familiarise themselves with each classification system. Respondents then undertook a warm-up rank task ranking 8 health states either in AQL-5D or in AQL-6D alongside 2 generic states ‘full health’ and ‘dead’. Respondents then completed a practise TTO question for a separate state followed by TTO questions valuing all 8 health states seen in the rank task. The protocol uses the York Measurement and Valuation of Health study version of TTO (Gudex, 1994) including the visual prop with generic full health (not instrument specific full health). At the end of the interview, respondents were asked to complete questions covering their demographic and socio-economic characteristics. All interviews were conducted from October 2009 to January 2010.

### Analyses of preference data

TTO values were obtained using the conventional transformations for states better and worse than dead to ensure a potential range 1.0 to −1.0 (Dolan, 1997). Three exclusion criteria were applied to the data to remove those respondents that do not appear to understand the task. Rrespondents were excluded from the analyses for valuing all states as identical, except in cases where all states are valued at one. Valuing all states as equal to one may not reflect a lack of understanding, but rather an unwillingness to trade life years for better health states. A second exclusion criterion was where respondents valued the worst possible health state higher than every other state. Finally respondents were excluded who valued all states as worse than dead.

The impact of adding pain/discomfort to AQL-5D was examined in three ways. Firstly, the mean values for the matched states were compared. Secondly, TTO values were modelled and the 5 asthma-specific coefficients were compared across AQL-5D and AQL-6D. Thirdly, the significance of the pain coefficients in the AQL-6D model was examined.

#### Comparisons of health state values

Mean health state values of the 4 matched health state pairs (e.g. 33333 and 333333) were compared using independent samples *t*-tests.

#### Modelling

Regression analysis is used to estimate the disutility associated with each level of each dimension, in order to enable utility scores to be estimated for all health states described by the classification system. Models have been estimated for the AQL-5D and the AQL-6D using ordinary least squares and a random effects component to allow for repeated health state values from the same respondent. Given the limitations of the study design and sample size it was only possible to estimate additive models. The standard random effects specification is (Brazier, Roberts, & Deverill, 2002):

(1) $$\left(1-y_{ij}\right)=\alpha +\beta x_{i}\mathrm{+\delta}z_{j}+\varepsilon_{ij}$$

where *i* = 1,2,…,*n* represents health states and *j* = 1,2,…,*m* represents respondents. The dependent variable $1-y_{ij}$ is TTO disvalue, where $y_{ij}$ is the TTO value for health state *i* valued by respondent *j*, $x_{i}$ is a vector of dummy explanatory variables for each level *λ* of dimension δ of the health state classification where level *λ* = 1 acts as a baseline for each dimension, and $z_{j}$ is a vector of socio-demographic characteristics. $\varepsilon_{ij}$ is the error term, subdivided into $\varepsilon_{ij}=u_{j}+e_{ij}$, where *u_j_* is the individual random effect and *e_ij_* is the usual random error term. This specification assumes a simple additive functional form. The data set is not designed to formally examine interactions within the AQL-5D, however we did examined an ‘N3′ dummy variable to pick-up possible interactions between the worst levels across the dimensions. It assumes a value of one when any dimension is at the worst level

TTO data is notoriously non-normal and associated with being skewed, censored at 1 and having more than one mode. The left skew in this data, whereby 25% of the values lie between 0.9 and 1, can be accounted for in a Tobit model with upper censoring, which treats the data as if they arise from a censored observation mechanism through which observations with true values greater than one are observed as 1.

Model performance was assessed in terms of adjusted R squared (where available), the likelihood ratio and the size and significance of individual parameter estimates. Predictive ability was assessed by the individual level root mean square error (RMSE) and the mean absolute error at the state level (i.e. the difference between predicted and actual mean values at the state level). Plots were used to illustrate possible patterns of predicted errors. The only difference between the AQL-5D and AQL-6D models is the addition of an extra pain/discomfort dimension. The coefficients on the non-pain dimensions of the models were compared using the *z*-score test for each dimension, where an absolute *z*-score of 1.96 or more would indicate a significant difference at the 5% level.

### Application of the valuation results to clinical trial data

To understand the practical implications of the findings of this study the regression models for the AQL-5D and AQL-6D were applied to clinical trial data. If the impact of an additional pain/discomfort dimension is entirely independent and additive, then health state scores of real asthma patients using AQL-6D will either be equal to or worse than the scores of the same patients using AQL-5D, since the additional information captured by AQL-6D is either neutral (no pain) or worse (moderate or extreme pain/discomfort).

The COGENT study at the University of Newcastle was a before and after, cluster randomised controlled trial, the objective of which was to evaluate the use of computerised decision support (CDS) systems in implementing clinical guidelines for the primary care management of asthma in adults (Eccles et al., 2000). UK practises which used their computer systems intensively were eligible for the study. Asthma patients aged 18 and over who were registered with the participating practises were identified from a computerised search. Patient-reported outcome questionnaires, including generic measures EQ-5D and SF-36, as well as asthma-specific measures NASQ (the Newcastle Asthma Symptom Questionnaire, Eccles et al. 2000) and AQLQ were administered in 3 rounds approximately 1 year apart. The analysis reported here uses round 1 data (*n* = 3059) but includes only observations with no missing data across all items required to produce an AQL-6D score (*n* = 2791). The AQL-6D is constructed from the AQLQ and EQ-5D. Mean age of the sample is 48.07 years (s.d. = 17.60) and 60.01% are female. For further details of the study, see Eccles et al (2000).

Values generated by the AQL-5D and AQL-6D will be compared in terms of mean scores for the whole sample and for sub-samples grouped by asthma symptom scores and pain/discomfort level.

## The valuation data

The response rate for all eligible respondents answering their door at time of interview was 45.8%. Respondents were excluded from the analyses for valuing all states as identical and less than one; valuing the worst possible health state higher than every other state; or valuing all states as worse than dead. This resulted in the exclusion of just 2 respondents out of 184 successfully conducted interviews.

The characteristics of the respondents were comparable to those of South Yorkshire and the UK for age and gender, but tended to have a higher proportion of retired individuals, a lower proportion of employed individuals and a lower mean EQ-5D score (0.80 vs. 0.86) (Table 2). There were no significant differences between the samples who valued the AQL-5D and AQL-6D in terms of age, gender, employment, education and health (Table 3).

There were 1455 TTO values elicited from 180 respondents with 727 and 728 for the AQL-5D and the AQL-6D health states respectively. Descriptive statistics across the health states are presented in Table 4. Three pairs of matched states were each valued between 60 and 62 times, the worst states (33333 and 333333) were valued 91 times each and the remaining states were valued between 29 and 31 times.

Across all health states TTO values range from −0.98 to 1.0, with 34.3% and 29.5% of observations having a value of 1.0 for ALQ-5D and AQL-6D respectively. Mean values for the 35 health states ranged from 0.26 to 0.98 and are generally lower than median values reflecting the negatively skewed distribution. Standard deviations were quite high and ranged from 0.07 to 0.58 and are comparable to those found in the original valuation of the AQL-5D.

## Results

### Comparison of health state values

Across the 4 matched states, mean values for the best (0.97 vs. 0.98) and worst states (0.26 vs. 0.30) of AQL-5D and AQL-6D were not found to be significantly different (Table 4). The mean value of 12132 from the AQL-5D (0.70) was significantly higher than 121323 from AQL-6D (0.56) (*p*-value = 0.061). By way of contrast, the mean value for the AQL-5D state 23131 (0.64) was lower than the AQL-6D state 231311 (0.78) (*p*-value = 0.034).

### Modelling of the preference data

There are four models presented in Table 5: OLS (models (1) and (3)) and random effects models (models (2) and (4)) for the AQL-5D and AQL-6D descriptive systems. The OLS and random effects models are quite similar, so the remaining presentation focuses on the latter.

**Fig. 1:**
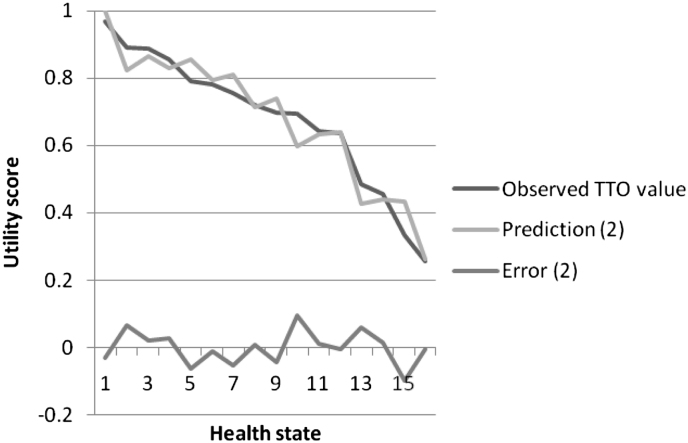
Observed and predicted values for AQL-5D model (2).

For AQL-5D model (2) the coefficients across the 5 dimensions are consistent with the severity levels within each dimension i.e. coefficients for level 3 > level 2 > level 1. The only exception is the sleep dimension, where the level 2 coefficient has the ‘wrong’ sign though it is very small and non-significant. Level 3 of breath, weather and sleep are significant as are levels 2 and 3 for activities. The RMSE at the individual level is quite high at 0.398, but the MAE at the state level is only 0.038 and this compares very favourably with that achieved in the original model of 0.047 (Yang et al, 2011). The plot of observed and predicted mean health state TTO values and residuals ordered by mean observed value suggests there is no obvious pattern in the errors (Fig. 1). The N3 term was not significant in any model.

For AQL-6D the pain/discomfort dimension had significant coefficients for levels 2 and 3 at the 5% level, with level 3 pain/discomfort having the largest coefficient (0.301) of any dimension in the AQL-6D for model (4). There were 3 inconsistencies with levels 2 of breath (−0.001), weather (−0.016), and sleep (−0.001) being negative, but these are all below 0.02 and none were significant. Overall the model performed well in terms of MAE (0.030 vs. 0.038 for AQL-5D) at the state level and again there is no obvious pattern in the errors. There was little change to the coefficients for concern and sleep compared to the AQL-5D model, but a noticeable reduction in the coefficient for level 3 of weather (which was significant in the AQL-5D model at the 5% level but non-significant in the AQL-6D model). However, there were substantial reductions in the coefficients for shortness of breath and activities, particularly for the level 3 coefficients of 0.167 vs. 0.047 and 0.307 vs. 0.150 for the AQL-5D and AQL-6D models respectively. The results of the *z*-tests confirm that there were significant differences between the AQL-5D and AQL-6D models in the coefficients of level 3 for shortness of breath and activities at the 1% level.

The marginal results for the Tobit model are reported in Table 6. They are entirely consistent for the AQL-5D with RE (model (6)) and there is only one inconsistency, namely one very small negative value (weather level 2) for AQL-6D in model (8). The Tobit models performance is slightly lower than the other models in terms of RMSE and MAE. The Tobit models, however, show the same key results: the levels for the add-on pain/discomfort dimension have significant coefficients; the coefficients for pain/discomfort at level 3 are the largest across all dimensions; in comparison to the AQL-5D models there are substantial and significant reductions in the level 3 coefficients for shortness of breath and activities.

### Application to clinical trial data

Models (2) and (4) in Table 5 have been applied to the COGENT trial data set using patient level completed AQLQ and EQ-5D data (Table 7). AQL-5D is derived from the AQLQ data and AQL-6D also uses the pain/discomfort dimension of the EQ-5D. Mean health state values produced using the AQL-5D are typically lower than the values produced using the AQL-6D by 0.1. The AQL-5D value is consistently lower than the AQL-6D value across the 5 asthma symptom severity groups, with the most severe asthma group having the largest difference.

**Fig. 2:**
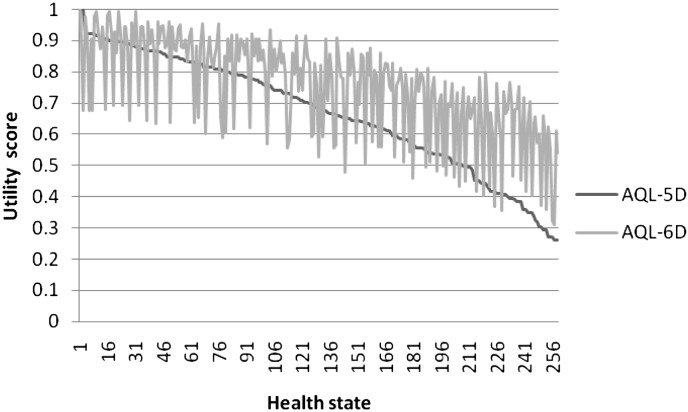
AQL-5D and AQL-6D by health state (*n* = 260 for AQL-6D) in patient data set.

To better understand the impact of the pain/discomfort dimension, the data set was divided into those reporting extreme, moderate and no pain using the EQ-5D pain/discomfort dimension. Except for the extreme pain/discomfort group, mean AQL-5D scores continue to be lower than mean AQL-6D scores, regardless of asthma symptom severity. For those with extreme pain/discomfort, AQL-5D scores are higher. Amongst this group, milder asthma symptoms are associated with larger differences between the AQL-5D and AQL-6D scores, but the 2 instruments result in very similar values for those with very severe asthma symptoms and extreme pain/discomfort. However, the numbers are low for some of the groups since only 6% of the sample report extreme pain/discomfort. The general pattern of AQL-6D exceeding AQL-5D is reflected in Fig. 2, where the predictions are ordered by AQL-5D health state value.

## Discussion

The health economics literature has tended to focus on the issues surrounding the valuation of health states, such as which valuation technique and whose values, rather than the role of the descriptive system. This study shows quite clearly that the content of the health state classification plays an enormous role in determining the values that are generated. The addition of the pain/discomfort dimension generated not only significant coefficients in its own right, but also had a significant impact on the coefficients of other dimensions. The adding-on of pain/discomfort was not simply additive in its impact on health state values.

Some degree of preference interaction has been shown to exist in a number of generic preference-based measures. For the HUI2 and HUI3 measures, a constant ‘k’ term was included in a multiplicative function that is consistent with preference complementarity (Feeny, Furlong, Torrance, & et al, 2002). For exposition purposes, imagine a set of health states where all other dimensions are at the best level, then preference complementarity is where the sum of the disutility associated with being in the bottom level of one dimension (say pain) and the disutility with being at the bottom of another dimension (say mobility) is less than the disutility associated with being at the bottom of these two dimensions together (Feeny, 2002). Evidence against a simple linear addition of main effects was also found in the significance of the additive ‘N3’ term for the EQ-5D and ‘MOST’ term for the SF-6D, which provided extra disutility where one of the dimensions was at its worse level, though these are not interaction terms in themselves (Brazier et al., 2002; Dolan, 1997). However, our study shows that the impact of the additional dimension for pain/discomfort is not consistent across dimensions and that a simple additive adjustment such as the N3 component of the UK EQ-5D value set or a constant multiplicative term as used for the Canadian valuation of the HUI2 and HUI3 would not adequately capture the effect of adding pain/discomfort. This has important implications for the use of measures, condition-specific or generic, that exclude important dimensions.

Another important result has been the way that adding a pain/discomfort dimension to the descriptive system has increased the value of the health state of most asthma patients in the COGENT trial except for those 6% with extreme pain/discomfort. This is also reflected in the results for the matched pairs where adding pain/discomfort at level 3 to 12132 reduces the values, as might be expected, but adding pain/discomfort at level 1–23131 has significantly increased the health state value. There was a similar finding in a study adding an additional sleep dimension to EQ-5D, where adding sleep level 1 to one of the two states significantly increased the mean health state value (0.179–0.486), though it did not alter the value of any other matched EQ-5D states (Yang et al, 2008). AQL-6D values even exceed those for AQL-5D in those with level 2 pain/discomfort in the COGENT study.

These apparently paradoxical results can be explained in a number of different ways. At least some respondents valuing the AQL-5D state may assume that the state being valued may involve pain/discomfort compared to respondents valuing the AQL-6D being told the state does not involve pain/discomfort. The ambiguity lies in the word discomfort and what has been read into this word by respondents. This may explain why AQL-5D scores are lower than AQL-6D scores for corresponding states with no pain/discomfort. In order to explain why AQL-5D scores are still lower than AQL-6D scores with moderate pain/discomfort, the magnitude of the unmentioned but imagined pain/discomfort needs to be quite substantial. Another explanation is that respondents may focus on one dominant dimension as part of a heuristic to simplify the task. For AQL-5D this is breathlessness or activities and for AQL-6D this for many may become pain/discomfort. This is related to the focussing effect mentioned earlier where respondents exaggerate the importance of asthma related problems, but the addition of the pain/discomfort dimension with no problems helps put those asthma problems into perspective and so they become less important (as reflected in the lower weights). Finally, there may be a simple explanation based on a simple heuristic, such as counting the number of level 1s and so adding level 1 pain/discomfort makes the state look better. Addressing these types of questions is better probed using more in-depth methods, such as cognitive de-briefing. Whatever the explanation, these all raise serious concerns about missing dimensions from any health state descriptive system.

There are a number of weaknesses to take into account with this study. Firstly, it was only possible to design a study to estimate additive functional forms similar to those that already exist for the EQ-5D. It would have been desirable to have estimated more complex functional forms such as multiplicative or multi-linear functional (Keeney and Raiffa, 1993). This was a consequence of funding limitations, but it was adequate for answering the primary question of whether adding a dimension to a descriptive system impacted on the size of the coefficients associated with other dimensions (including significant changes), though probably not for generating results for a new extended CSPBM. Secondly, the MVH TTO protocol can be criticised for using a 10 year time frame that is unrealistic in younger populations. It was selected due the evidence on its reliability in the general population and given this is a methodological study, it should not have any major implications for the findings of the study. Thirdly, the subject of the study has been one condition-specific instrument and this may limit the generalisability of the results to other CSPBMs or perhaps more importantly for policy makers, to generic preference-based measures. The results will be specific to asthma and the addition of pain/discomfort in particular, but CSPBMs by definition tend to exclude many common and important domains and so the general issue addressed by this study is relevant. Even generic measures exclude potentially important dimensions such as cognition and energy in the EQ-5D. However, whether other dimensions would have such a strong impact as pain/discomfort remains to be seen.

This study has found that adding a common and generic dimension to a CSPBM has an impact on the decrements associated with the dimensions specific to the condition. Comparability between measures (condition-specific or generic), requires among other things that the impact of different dimensions on preferences is additive, whether or not they are included in the classification system. Our results cast doubt on this assumption for important dimensions like pain/discomfort.

## Figures and Tables

**Table 1:** AQL-5D and AQL-6D classification system (3 level version).

Dimensions common to both measures
Concern about asthma
Feel concerned about having asthma none of the time
Feel concerned about having asthma some of the time
Feel concerned about having asthma all of the time
Shortness of breath
Feel short of breath as a result of asthma none of the time
Feel short of breath as a result of asthma some of the time
Feel short of breath as a result of asthma all of the time
Weather and pollution
Experience asthma symptoms as a result of air pollution none of the time
Experience asthma symptoms as a result of air pollution some of the time
Experience asthma symptoms as a result of air pollution all of the time
Sleep
Asthma interferes with getting a good night’s sleep none of the time
Asthma interferes with getting a good night’s sleep some of the time
Asthma interferes with getting a good night’s sleep all of the time
Activities
Overall, not at all limited in any activity done due to asthma
Overall, moderate or some limitation in every activity done due to asthma
Overall, totally limited in every activity done due to asthma

Pain and discomfort (Sixth dimension included in AQL-6D only)
Have no pain or discomfort
Have moderate pain or discomfort
Have extreme pain or discomfort

**Table 2:** Respondent characteristics.

	Sample (*n* = 182)	South Yorkshire a	England a
Mean age (s.d.)	51.07 (17.39)	NA	NA
Age distribution			
18–40	31.3%	41.2%	41.6%
41–65	42.9%	39.1%	39.1%
Over 65	25.8%	19.7%	19.3%
Female	60.4%	51.2%	51.3%
Married/Partner	73.1%	NA	NA
Main activity			
Employed or self-employed	33.0%	56.1%	60.9%
Unemployed (or seeking work)	8.7%	4.1%	3.4%
Long-term sick	6.0%	7.7%	5.3%
Full-time student	2.2%	7.5%	7.3%
Retired	32.4%	14.4%	13.5%
Own home outright or with a mortgage	78.0%	64.0%	68.7%
Renting property	22.0%	36.0%	31.3%
Secondary school is highest level of education	44.0%	NA	NA
EQ-5D score (s.d.)	0.80 (0.26)	NA	0.86 (0.23)^b^

a Statistics for South Yorkshire Health Authority and for England in the Census 2001. Questions used in this study and the census are not identical. The census includes persons aged 16 and above whereas this study only surveys persons aged 18 and above. Age distribution is here reported as the percentage of all adults aged 18 and over.

b Interviews conducted in the Measurement and Valuation of Health (MVH) study in 1993 (Gudex et al, 1994).

**Table 3:** Respondent characteristics for AQL-5D and AQL-6D.

	AQL-5D (*n* = 91)	AQL-6D (*n* = 91)	P-value^a^
Mean age (s.d.)	52.13 (17.54)	50.01 (17.27)	0.412^b^
Age distribution			
18–40	27.5%	35.2%	0.263
41–65	46.2%	39.6%	0.369
Over 65	26.4%	25.3%	0.866
Female	61.5%	59.3%	0.762
Married/partner	70.3%	75.8%	0.403
Main activity			
Employed or self-employed	30.8%	35.2%	0.528
Unemployed (or seeking work)	11.0%	6.6%	0.295
Long-term sick	3.3%	8.8%	0.120
Full-time student	3.3%	1.1%	0.310^c^
Housework	9.9%	15.4%	0.265
Retired	36.3%	28.6%	0.268
Own home outright or with a mortgage	76.9%	79.1%	0.720
Renting property	23.1%	20.9%	
Secondary school is highest level of education	46.2%	41.8%	0.550
Doubtful whether respondent understood TTO (interviewer reported)	2.2%	1.1%	
Have asthma	25.3%	23.1%	0.729
Have moderate pain or discomfort	39.6%	30.8%	0.214
Have extreme pain or discomfort	4.4%	8.8%	0.232
EQ-5D score (s.d.)	0.79 (0.25)	0.80 (0.28)	0.850^b^
Time taken	31.85 (8.24)	31.62 (8.89)	0.856^b^

a Pearson Chi-square *p*-value.

b ANOVA *p*-value.

c Fisher’s exact test *p*-value (2-sided) as the expected frequency is 5 or lower.

**Table 4:** Health state values for AQL-5D and AQL-6D.

Measure	Health state	n	Mean (s.d.)	Median	IQR	Min	Max
AQL-5D	**11111^a^**	60	0.97 (0.14)	1.00	1.00–1.00	0.03	1.00
	11231	31	0.75 (0.42)	0.93	0.70–1.00	−0.98	1.00
	11321	60	0.89 (0.18)	1.00	0.83–1.00	0.03	1.00
	12123	29	0.69 (0.31)	0.78	0.44–0.97	−0.30	1.00
	**12132^b^**	62	0.70 (0.41)	0.78	0.64–1.00	−0.98	1.00
	13213	31	0.46 (0.58)	0.63	0.03–0.93	−0.93	1.00
	13312	62	0.64 (0.50)	0.80	0.50–1.00	−0.98	1.00
	21222	60	0.78 (0.33)	0.90	0.66–1.00	−0.98	1.00
	22311	30	0.86 (0.21)	0.93	0.79–1.00	0.13	1.00
	23113	31	0.33 (0.56)	0.50	0.03–0.78	−0.98	1.00
	**23131^c^**	60	0.64 (0.44)	0.80	0.54–0.95	−0.98	1.00
	31112	29	0.89 (0.15)	0.95	0.83–1.00	0.40	1.00
	31333	29	0.49 (0.37)	0.50	0.30–0.76	−0.48	1.00
	32211	31	0.79 (0.44)	1.00	0.78–1.00	−0.98	1.00
	33121	31	0.72 (0.33)	0.75	0.53–1.00	−0.28	1.00
	**33333^d^**	91	0.26 (0.53)	0.33	0.00–0.63	−0.98	1.00

AQL-6D	**111111^a^**	61	0.98 (0.07)	1.00	1.00–1.00	0.50	1.00
	112322	31	0.77 (0.21)	0.80	0.60–1.00	0.35	1.00
	**121323^b^**	60	0.56 (0.40)	0.55	0.36–0.89	−0.70	1.00
	123212	30	0.84 (0.20)	0.93	0.78–1.00	0.28	1.00
	132231	30	0.86 (0.23)	0.98	0.79–1.00	0.00	1.00
	133133	31	0.40 (0.46)	0.50	0.00–0.83	−0.88	1.00
	211232	30	0.69 (0.23)	0.70	0.47–0.89	0.20	1.00
	213333	30	0.44 (0.52)	0.50	0.25–0.91	−0.93	1.00
	222213	31	0.66 (0.32)	0.73	0.50–0.93	−0.23	1.00
	223121	30	0.90 (0.19)	1.00	0.90–1.00	0.13	1.00
	**231311^c^**	61	0.78 (0.24)	0.90	0.54–1.00	0.10	1.00
	232122	30	0.79 (0.20)	0.83	0.64–1.00	0.28	1.00
	312113	30	0.65 (0.35)	0.73	0.49–0.94	−0.48	1.00
	313221	31	0.83 (0.17)	0.85	0.75–1.00	0.30	1.00
	321132	30	0.78 (0.25)	0.85	0.53–1.00	0.03	1.00
	322331	30	0.68 (0.33)	0.74	0.48–0.93	−0.48	1.00
	331223	31	0.52 (0.44)	0.63	0.25–0.85	−0.93	1.00
	333312	30	0.73 (0.23)	0.76	0.56–0.93	0.23	1.00
	**333333^d^**	91	0.30 (0.48)	0.33	0.00–0.65	−0.98	1.00

Notes: Matched health states (those with 5 shared dimensions) across both studies are in bold. Results of independent *t*-test comparing matched states: a. *p* = 0.492, b. *p* = 0.061, c. 0.034, d. 0.576.

**Table 5:** Regression analysis estimating values sets for AQL-5D and AQL-6D.

	AQL-5D		AQL-6D		Z-score	
	(1)	(2)	(3)	(4)	(1) v (3)	(2) v (4)
Concern2	0.039	0.032	0.031	0.034	0.170	−0.052
Concern3	0.035	0.041	0.046	0.047∗∗	−0.210	−0.165
Breath2	0.042	0.019	−0.011	−0.001	0.984	0.502
Breath3	0.200∗∗∗	0.167∗∗∗	0.054∗	0.047∗	2.853∗∗∗	3.177
Weather2	0.069	0.024	−0.015	−0.016	1.599	1.035
Weather3	0.058	0.057∗∗	0.034	0.033	0.513	0.734
Sleep2	−0.001	0.016	0.017	−0.001	−0.346	0.471
Sleep3	0.121∗∗∗	0.106∗∗∗	0.099∗∗∗	0.091∗∗∗	0.471	0.431
Activities2	0.080∗∗	0.074∗∗∗	0.042	0.040∗	0.795	0.978
Activities3	0.290∗	0.307∗∗∗	0.137∗∗∗	0.150∗∗∗	2.921∗∗∗	4.213∗∗∗
Pain2			0.071∗∗	0.071∗∗∗		
Pain3			0.303∗∗∗	0.301∗∗∗		

Constant	0.034	0.061	0.019	0.023	0.260	0.681

Observations	727	727	728	728		
Number of id		91		91		
R-squared	0.223		0.280			
Root MSE	0.398	0.398	0.323	0.323		
MAE (state level)	0.031	0.038	0.027	0.030		

*Notes:* ∗ significant at 10%; ∗∗ significant at 5%; ∗∗∗ significant at 1%.

**Table 6:** Tobit regression analysis estimating values sets for AQL-5D and AQL-6D.

	AQL-5D				AQL-6D				Z-score	
	(5)	Marginal effects	(6)	Marginal effects	(7)	Marginal effects	(8)	Marginal effects	(5) V (7)	(6) V (8)
Concern2	0.042	0.039	0.039∗	0.035∗	0.031	0.029	0.031	0.029	0.270	0.260
Concern3	0.042	0.039	0.045∗∗	0.042∗∗	0.046∗	0.044∗	0.045∗∗	0.042∗∗	−0.102	0.015
Breath2	0.035	0.032	0.021	0.020	−0.006	−0.006	0.003	0.003	0.940	0.578
Breath3	0.163∗∗∗	0.150∗∗∗	0.143∗∗∗	0.131∗∗∗	0.057∗∗	0.054∗∗	0.049∗∗	0.046∗∗	2.552∗∗	2.961∗∗∗
Weather2	0.045	0.041	0.016	0.015	−0.010	−0.009	−0.007	−0.007	1.274	0.740
Weather3	0.050∗	0.046∗	0.049∗∗	0.045∗∗	0.029	0.027	0.027	0.026	0.538	0.783
Sleep2	0.018	0.017	0.028	0.025	0.019	0.018	0.002	0.002	−0.032	0.820
Sleep3	0.117∗∗∗	0.108∗∗∗	0.106∗∗∗	0.097∗∗∗	0.091∗∗∗	0.086∗∗∗	0.080∗∗∗	0.075∗∗∗	0.676	0.908
Activities2	0.069∗∗	0.064∗∗	0.066∗∗∗	0.060∗∗∗	0.035	0.033	0.035∗	0.033∗	0.858	1.086
Activities3	0.264∗∗∗	0.236∗∗∗	0.274∗∗∗	0.244∗∗∗	0.124∗∗∗	0.116∗∗∗	0.140∗∗∗	0.130∗∗∗	3.284∗∗∗	4.282∗∗∗
Pain2					0.073∗∗	0.069∗∗	0.068∗∗∗	0.063∗∗∗		
Pain3					0.280∗∗∗	0.260∗∗∗	0.275∗∗∗	0.254∗∗∗		

Constant	0.032		0.056		0.023		0.035		0.204	0.444

Observations	727		728		727		729			
Number of id					91		92			
Pseudo R-squared	0.322				0.418					
Root MSE	0.404		0.404		0.326		0.326			
MAE (state level)	0.059		0.062		0.041		0.042			

*Notes:* ∗ significant at 10%; ∗∗ significant at 5%; ∗∗∗ significant at 1%.

**Table 7:** Application of AQL-5D and AQL-6D to a patient data set (*n* = 2791).

	All patients			No pain/discomfort in EQ-5D			Moderate pain/discomfort in EQ-5D			Extreme pain/discomfort in EQ-5D		
	AQL-5D	AQL-6D		AQL-5D	AQL-6D		AQL-5D	AQL-6D		AQL-5D	AQL-6D	
	Mean (s.d.)	Mean (s.d.)	n	Mean (s.d.)	Mean (s.d.)	n	Mean (s.d.)	Mean (s.d.)	n	Mean (s.d.)	Mean (s.d.)	n
All patients	0.733 (0.188)	0.833 (0.144)	2791	0.817 (0.119)	0.930 (0.060)	1245	0.677 (0.198)	0.787 (0.108)	1373	0.563 (0.226)	0.495 (0.126)	173
Asthma symptoms score												
0 < NASS^a^≤20 (least severe)	0.884 (0.087)	0.933 (0.069)	625	0.892 (0.089)	0.962 (0.040)	446	0.864 (0.083)	0.878 (0.035)	164	0.871 (0.071)	0.649 (0.025)	15
20 < NASS≤40	0.808 (0.083)	0.893 (0.072)	528	0.817 (0.078)	0.933 (0.043)	302	0.795 (0.088)	0.853 (0.045)	214	0.803 (0.065)	0.621 (0.041)	12
40 < NASS≤60	0.759 (0.114)	0.859 (0.090)	583	0.781 (0.093)	0.915 (0.052)	267	0.742 (0.128)	0.828 (0.065)	295	0.721 (0.137)	0.589 (0.078)	21
60 < NASS≤80	0.697 (0.149)	0.805 (0.122)	478	0.743 (0.122)	0.897 (0.067)	155	0.680 (0.156)	0.791 (0.086)	285	0.637 (0.155)	0.538 (0.087)	38
80 < NASS≤100 (most severe)	0.489 (0.189)	0.655 (0.157)	539	0.624 (0.155)	0.831 (0.091)	64	0.486 (0.187)	0.681 (0.106)	389	0.401 (0.163)	0.407 (0.099)	86

*Notes:*^a^. NASS is the Newcastle Asthma Symptoms Score.
